# Current Approaches and Applications in Avian Genome Editing

**DOI:** 10.3390/ijms21113937

**Published:** 2020-05-30

**Authors:** Joonbum Lee, Dong-Hwan Kim, Kichoon Lee

**Affiliations:** 1 Department of Animal Sciences, The Ohio State University, Columbus, OH 43210, USA; lee.3920@osu.edu (J.L.); kim.4094@osu.edu (D.-H.K.); 2 The Ohio State University Interdisciplinary Human Nutrition Program, The Ohio State University, Columbus, OH 43210, USA

**Keywords:** avian, genome editing, CRISPR/Cas9, primordial germ cell (PGC), adenovirus

## Abstract

Advances in genome-editing technologies and sequencing of animal genomes enable researchers to generate genome-edited (GE) livestock as valuable animal models that benefit biological researches and biomedical and agricultural industries. As birds are an important species in biology and agriculture, their genome editing has gained significant interest and is mainly performed by using a primordial germ cell (PGC)-mediated method because pronuclear injection is not practical in the avian species. In this method, PGCs can be isolated, cultured, genetically edited in vitro, and injected into a recipient embryo to produce GE offspring. Recently, a couple of GE quail have been generated by using the newly developed adenovirus-mediated method. Without technically required in vitro procedures of the PGC-mediated method, direct injection of adenovirus into the avian blastoderm in the freshly laid eggs resulted in the production of germ-line chimera and GE offspring. As more approaches are available in avian genome editing, avian research in various fields will progress rapidly. In this review, we describe the development of avian genome editing and scientific and industrial applications of GE avian species.

## 1. Introduction

Genome editing contributes to advances in many fields of biology by changing DNA sequences for desired phenotypic traits. To conduct targeted genome editing, a method using homologous recombination was first discovered [1]. Homologous recombination occurs in a cell after introducing long DNA fragments containing homologous DNA sequence as a wing tag, which can recombine with the target genome in the cell to replace the targeted region. However, the efficiency of homologous recombination was extremely low in most cell types, and inaccurate insertion of the exogenous DNA fragment into an unintended region of the genome was another problem. To improve the efficiency and accuracy of conventional gene targeting, a new method relying on site-specific nucleases (SSNs), such as zinc-finger nucleases (ZFNs) and transcription activator-like effector nucleases (TALENs), was developed [2,3]. SSNs can induce a double-stranded break (DSB) after recognizing and binding to a specific DNA sequence and generate random insertion or deletion (indel) mutations by repairing DSB with error-prone non-homologous end joining (NHEJ), or by homologous direct repair (HDR), by providing a donor vector as a repair template [4]. Although ZFNs and TALENs had been mainly used for genome editing as early SSNs, these technologies are hampered by low efficiency due to short recognition length (ZFNs: 5 to 7 bp and TALENs: 12 to 20 bp) and difficulties to assemble [4,5]. To overcome the limitation, the clustered regularly interspaced short palindromic repeats (CRISPR)/Cas9 system was developed as the most powerful and efficient SSNs and greatly improved genome-editing technologies [6]. Compared to ZFNs and TALENs, the CRISPR/Cas9 system has higher efficiency and specificity because the CRISPR/Cas9 recognizes a longer nucleotide spacer region (20 bp) of guide RNA (gRNA), and the system enables rapid construction of the vector because only gRNA needs to be replaced in the backbone vector for a specific target [5].

In addition to the advance in genome-editing technologies, availability of a large number of sequenced animal genomes enables targeted genome editing in livestock animals [7]. Various genome-edited (GE) livestock can serve as advantageous animal models in scientific and biomedical research due to their physiological compatibility with humans. Such livestock animals include cattle for studying human female reproduction; sheep for studying human lung function and disease; and pigs for studying various human diseases, including diabetes, muscular dystrophy, immunodeficiency, and cancer [8]. Furthermore, desirable traits of livestock animals for industrial benefit are obtained through genome editing, such as increased milk and wool production from GE cows and sheep [7]. In this regard, genome editing in the avian species gains a significant interest to benefit both biological research and the poultry industry. In spite of the attention and importance in avian genome editing, however, the progress in avian genome editing is relatively slow compared to mammalian genome editing. In this review, we summarize limitations, current approaches, and applications in avian genome editing.

## 2. Limitation of One-Cell Embryo Microinjection in Avian Genome Editing

Since the introduction of the pronuclear injection in the early 1980s, microinjection of genetic materials or genome-editing tools into the embryo at the one-cell stage, called zygote, becomes the major technique for generation of transgenic or GE mammals and fishes [9,10,11,12,13]. By modifying or editing the genome of zygote, whole-body genome editing can be obtained within a single generation. However, such a strategy has not been successfully applied in avian genome editing because of differences in the reproductive system and embryonic development. In this section, we summarize limitations of the avian reproductive system for genome editing via microinjection approach.

### 2.1. Before Ovulation

**Figure 1:**
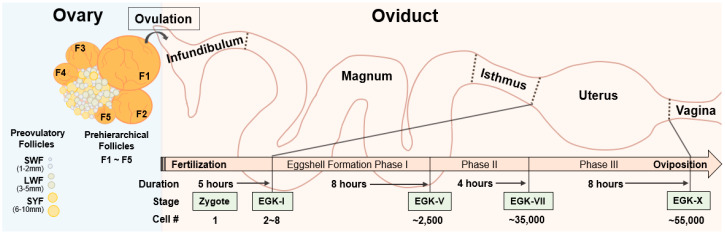
Overview of the reproductive system and embryonic development in chicken. Avian ovary contains large number of preovulatory follicles, small white follicles (SWF, 1–2 mm), large white follicles (LWF, 3–5 mm), and small yellow follicles (SYF, 6–10 mm), and approximately five prehierarchical follicles, naming F1 to F5, depending on their sizes. After ovulation, F1 follicles enters the avian oviduct and fertilizes in the infundibulum. After passing magnum and isthmus, a one-cell zygote starts first cleavage in the uterus and becomes an Eyal-Giladi and Kochav stage (EGK)-X blastoderm, having approximately 55,000 cells at the time of oviposition.

For pronuclear injection in mammals, fertilized oocytes can be obtained by hormonal induction of ovulation and followed by mating, and the oocytes can then be genetically edited via microinjection of genome-editing tools and transferred into a pseudopregnant animal, to generate a targeted GE offspring [14]. However, fertilized oocytes are difficult to obtain in avian species due to their unique development and maturation process. In the chicken, oocytes undergo three growth phases: initial, intermediate, and rapid growth phase [15]. During the initial growth phase, oocytes become primordial follicles, which are quiescent for months or years. After a slow and long initial growth phase, the primordial follicles become the visible primary follicles in the ovaries. The ovaries of sexually matured birds contain different sized follicles which can be largely divided into two groups: prehierarchical follicles and preovulatory follicles [16,17]. Prehierarchical follicles are composed of small white follicles (SWF, 1–2 mm), large white follicles (LWF, 3–5 mm), and small yellow follicles (SYF, 6–10 mm), and they are in the intermediate growth phase for approximately two months [18]. Most of the LWF and SYF undergo atresia and reabsorption by the ovary, while one of the SYF is selected daily to be recruited into the yolk-filled preovulatory hierarchy [19]. Preovulatory follicles enter a rapid growth phase, and the oocyte from the largest preovulatory follicle (F1) among five to seven preovulatory follicles is ovulated, and the next largest preovulatory follicle (F2) becomes the largest sequentially (Figure 1). Therefore, maturation of an avian oocyte has to go along with the accumulation of yellow yolk, which is almost impossible to imitate artificially. Although the oocyte from the preovulatory follicles can be obtained, fertilized in vitro, and transferred to the oviduct of a recipient hen for egg development [20,21], the techniques are labor intensive and difficult to be used for production of GE birds. In addition, it will be hard to handle the fragile and soft yolk-filled part during the whole processes. Due to these difficulties, technology for avian transgenesis and genome editing did not depend on using preovulatory oocytes.

### 2.2. After Ovulation

Unlike complete embryonic development in the oviduct in mammals, avian oocytes are developed into a blastodermal stage, along with the formation of the egg in the oviduct. After ovulation in the chicken, the oocyte immediately enters the oviduct, which consists of five sections: infundibulum, magnum, isthmus, uterus, and vagina [22,23]. Shortly after ovulation, the oocyte is fertilized in the infundibulum, and the fertilized oocyte travels through the oviduct for accumulation of egg white surrounding the yolk in the magnum and formation of inner and outer eggshells in the isthmus. Then, calcification of the eggshell is carried out in three phases, followed by eggshell pigmentation and cuticle deposition, in the uterus (Figure 1) [24,25]. After completion of the egg formation, the eggs are laid through vagina and the whole process, from ovulation to oviposition, takes approximately 25 h.

After fertilization of ovulated oocyte in the infundibulum, the first cell division of one-cell zygote occurs in the uterus approximately five hours after ovulation, and the zygote develops into the Eyal-Giladi and Kochav stage (EGK)-I embryo [26,27,28]. The embryo stays in the uterus for approximately 20 h, when the cell numbers are increased exponentially from the EGK-I embryo, having 2–8 cells to the EGK-X blastoderm containing approximately 55,000 cells (Figure 1) [24,29]. Therefore, rapid development of a zygote in a complex avian oviduct makes it difficult to obtain one-cell zygotes. Although there is a time window of approximately 5 h for obtaining a one-cell zygote in the oviduct prior to the preovipositional development in the uterus by sacrificing a hen shortly after oviposition [28,30], development of the zygote in surrogated eggshells with artificial media is not efficient, showing approximately 7–35% of hatching rate [31,32], and transferring the fertilized ovum into the infundibulum of a recipient hen is technically difficult [20]. Therefore, sacrificing a hen for obtaining, genome editing, and developing a one-cell zygote into a chick is not practical.

## 3. Current Approaches for Avian Genome Editing

For avian genome editing, a primordial germ cell (PGC)-mediated method has been widely used and an adenovirus-mediated method has been recently introduced. Both methods have their own pros and cons that seem to complement each other. In this section, we review the two currently available avian genome-editing methods.

### 3.1. PGC-Mediated Method

**Figure 2:**
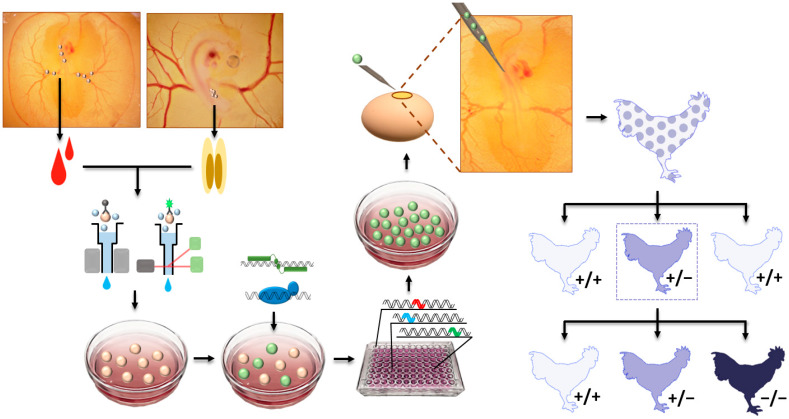
Graphical summary of primordial germ cell (PGC)-mediated method for genome editing in the chicken. Chicken PGCs are isolated from embryonic blood or gonad by magnetic activated cell or fluorescence-activated cells sorting, using PGC-specific antibodies. After culture of chicken PGCs, genome-editing tools, transcription activator-like effector nucleases or clustered regularly interspaced short palindromic repeats (CRISPR)/Cas9, are applied to cells which are screened by antibiotic selection or fluorescence-activated cell sorting. Then, single cells are proliferated and sequenced to detect genome-edited cells for injection into the dorsal aorta of recipient chicken embryos. After generation of potential germ-line chimeric chicken, the chimeras are mated with wild-type partners to produce wild-type (+/+) and heterozygous mutant (+/−) chickens. The +/− offspring are mated with another +/− offspring to generated +/+, +/−, and homozygous mutant (−/−) chickens.

As a previously used major technique for gene knockout, the embryonic stem cell (ESC)-mediated method has been used to produce knockout mice, because ESCs are pluripotent and germline-competent cells [1]. After delivery of knockout vectors, ESCs having a desired knockout are screened and injected to recipient mouse blastocysts, to produce germline chimaeras and consequent GE offspring. Because the efficiency of pronuclear injection of knockout vector for homologous recombination was low and desired knockout in ESCs can be prescreened, the ESC-mediated method was preferable for precise genome editing versus pronuclear injection before the advance in genome-editing technologies. In this regard, germline-competent stem cells, including ESCs [33,34], blastodermal cells [35,36,37], spermatogonial stem cells [38], embryonic germ cells (EGCs) [39,40], and PGCs [41,42,43,44,45,46,47,48], were used to generate germline chimeras and transgenic birds. In avian species, a relatively higher germline competency of PGCs compared to other stem cells, such as ESCs and EGCs [33,40,47], made it more attractive to cell types for avian transgenesis. Accordingly, the first GE chicken was generated via homologous recombination in chicken PGCs in vitro and, subsequently, injection of the PGCs with desired genome edition into recipient embryos [49]. Since then, TALENs- and CRISPR/Cas9-mediated genome editing in chickens have been performed [50,51]. As a precursor cell of gametes, PGCs settle into the genital ridge and differentiate into mature germ cells during embryonic development in mammals and birds [52]. However, mammalian PGCs migrate through dorsal mesentery within the body, whereas migration of avian PGCs is more dynamic because blastodermal PGCs initially migrate to the germinal crescent of the embryo, circulate through an extraembryonic blood vessel, and settle into the genital ridge [52]. In this regard, circulating or gonadal PGCs can be isolated from a different stage of embryos (Figure 2). After genome editing in cultured PGCs, the GE PGCs are injected into the blood vessel of recipient embryos, so that the exogenous GE PGCs can be settled into the genital ridge, along with endogenous PGCs, to eventually produce GE offspring (Figure 2). Furthermore, exogenous PGCs from different breeds of chicken can be injected into host embryos, in order to conserve and regenerate rare chicken breeds [53].

PGC-mediated avian genome editing has several advantages. First, prescreening of targeted GE PGCs is achievable, and GE birds with targeted mutation can be produced by injecting the prescreened GE PGCs. Moreover, the generation of knock-in birds is achievable because multiple vectors can be co-transfected, and PGCs having knock-in event can be screened [54,55]. In addition, if PGCs from colored birds were used and injected into a white recipient embryo, offspring originated from injected PGCs can be prescreened by feather color, thus reducing the cost for genotyping [56]. In this method, for GE PGCs to serve as a precursor of functional gametes in the gonad after injection, cultured PGCs have to retain their germ cell characteristics without differentiating into other cell types during the whole in vitro experimental procedures. However, isolation, culture, and genome modification of PGCs, as well as screening of GE PGCs, are long and complex processes (Figure 2) which require highly skilled techniques. Moreover, optimal conditions for PGC culture isolated from different avian species have to be established. Partially due to these difficulties, application of this method for in vivo avian genome editing is currently limited to chickens.

### 3.2. Adenovirus-Mediated Method

Injection of genetic materials into one of the pronuclei in the fertilized egg has been mainly used to generate transgenic animals. As an alternative method to increase efficiency of delivery and integration of transgene into the host’s genome, a lentiviral delivery system was used to infect cultured oocytes and generate transgenic animals [57,58,59,60,61]. Lentivirus is a class of retroviruses that can infect both dividing and nondividing cells and integrate a viral genome into the host’s genome [62]. The lentiviral gene delivery system was also primarily used to generate transgenic birds by the injection of lentivirus into avian blastoderm for inducing the integration of transgene into the genome of germline cells in the blastoderm [63,64,65,66,67,68]. However, an integration event of the lentivirus is a critical disadvantage in CRISPR/Cas9-mediated gene editing, because random integration of CRISPR/Cas9 expression cassettes can cause insertional mutagenesis [69] and permanent expression of gRNA, and Cas9 protein increase the risk of off-target mutations [70].

**Figure 3:**
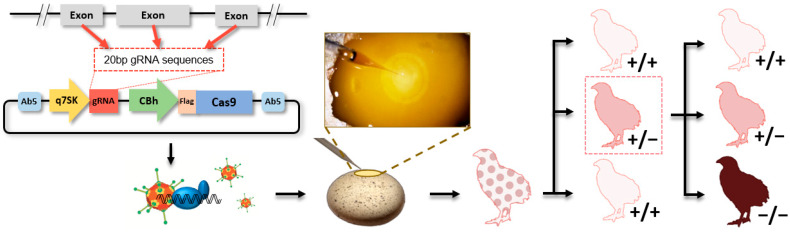
Graphical summary of adenovirus-mediated method for genome editing in quail. After designing guide RNA complement to target sequence in the exon of targeted gene, an adenovirus containing CRISPR/Cas9 system is produced. Then, the adenovirus is injected into the subgerminal cavity of quail blastoderm, and potential germ-line chimeric quail are produced. Subsequently, +/+ and +/− offspring are generated, and +/+, +/−, and −/− quail are produced from +/− parents.

To prevent the problems of lentivirus in CRISPR/Cas9-mediated genome editing, adenovirus containing the CRISPR/Cas9 system is injected into the quail blastoderm, to generate GE offspring (Figure 3) [71,72]. Unlike lentivirus, adenovirus episomally transduces the viral genome, without integration into the host genome and express foreign gene transiently, which precludes the risks of insertional mutagenesis from integration of the foreign gene and minimizes the risks of off-target mutation from constitutive expression of gRNA and Cas9 protein [70,73]. In addition, the adenovirus is able to transduce both dividing and quiescent cells, making it a suitable gene delivery system for highly proliferating blastodermal cells, and a large packaging capacity of the adenoviral vector makes it capable to insert a large fragment of gRNA and Cas9 expression cassettes (6–7 kb) in the adenoviral vector [73]. Moreover, a high titer of the adenovirus enables a smaller volume of the injection into the avian blastoderm, to reduce any damage from the injection. Adenoviral injection into the subgerminal cavity of avian blastoderm can directly edit the genome of PGCs located in the central region of the blastoderm, showing 2.4% to 10% of germline transmission efficiency for producing GE offspring [71,74]. Thus, genome editing in birds can be performed rapidly and conveniently by using the adenovirus-mediated method, without time-consuming and technically difficult steps, including PGC isolation, culture, in vitro genome edition, and injection of the GE PGC into the recipient embryos. Importantly, this method can potentially be applied to various avian species, as long as cells are receptive to adenoviral transduction, because adenovirus can be injected into any avian blastoderms. So far, transduction feasibility of adenovirus type 5 in chickens, quail, and turkey cells has been confirmed [71,75,76], and in vivo genome editing in quail, using the adenovirus-mediated method, has been reported [71,72]. However, limitations of the adenovirus-mediated method should also be considered. With this method, efficiency of knock-in would be very low, because required delivery of both the adenovirus containing the CRISPR/Cas9 system and the donor template into the same blastodermal PGCs, by injecting into blastoderm, will be very challenging. In addition, all offspring have to be genotyped, and GE offspring will have random indel mutation, because desired mutation cannot be prescreened at the cellular level.

## 4. Current Applications of Avian Genome Editing

Development of the CRISPR/Cas9 system enabled researchers to target specific genes easily, by changing gRNA. Such advances in genome-editing technologies also expand the application of avian genome editing in multiple sectors of biology, biomedical, and poultry industry (Table 1). In this section, we summarize currently reported avian models with targeted genome editing and their potential applications.

### 4.1. Scientific Purpose

Historically, the chicken has been an important vertebrate model in various fields of biology due to easy access of all developmental stages of embryos. Because avian and mammals have many common immunological systems, discoveries in avian immunology provided invaluable assistance for studying basic immunology in mammals [77]. Importantly, many features of lymphocytes were described in chickens, and antibody-producing B lymphocytes were first recognized in chickens [78]. To deeply study B-cell development and avian immunology, first GE-chicken-targeting immunoglobulin heavy chain (*IgH*) was generated via homologous recombination, using a PGC-mediated method [49]. Subsequently, immunoglobulin light chain (*IgL*) knockout chickens were also generated by using the same method [79]. Interestingly, *IgH* knockout in chickens blocks the development of B-cells, whereas *IgL* knockout showed a reduced population of B-cells. In addition, CRISPR/Cas9-mediated homologous recombination was performed to knockout *IgH* variable region and inserted a loxP site in the region [80]. In addition to studying avian immunology, the study of immunoglobulin locus can be potentially applied to the production of human antibody, by reconstructing the components of immunoglobulin.

In conventional avian genome editing, the chicken has been the most valuable model. However, quail can be a good alternative model in genome-editing study due to their small size, short generation time, and high level of egg production [81]. To conduct genome editing in quail, the adenovirus-mediated method was utilized, and the melanophilin (*MLPH*) gene was targeted, to show apparent phenotypic differences in feather color, because the *MLPH* gene is known to regulate coat and feather color in mammals and birds [71,82,83,84,85,86]. *MLPH* knockout quail also showed gray feathers and MLPH function in feather pigmentation, and other tissues can be investigated by using these GE quail. Furthermore, success of the adenovirus-mediated method opens a new stage of avian genome editing, because this method can be potentially applied to any type of avian species.

### 4.2. Industrial Purpose

In the broiler industry, traditional selection of breeders has focused on higher feed efficiency and faster growth rates [91]. In addition to the success of traditional selection, finding genetic markers for further improvements in growth rate, feed efficiency, and other desirable traits, such as disease resistance, has become significantly important. Thus, genome editing has been actively used to find genetic factors for desirable traits in various livestock species, including chickens. In the livestock industry, higher meat yield is one of the most important interests for economic profit. In this regard, the myostatin (*MSTN*) gene gains huge attention, because MSTN is a negative regulator of muscle growth, and mutation in the *MSTN* gene resulted in increased muscle mass in mammals and fishes [92,93,94,95,96,97,98,99,100,101,102]. To investigate anti-myogenic function of MSTN in the avian species, *MSTN* knockout quail and chickens were generated by using the adenovirus- and PGC-mediated method, respectively [72,87]. Significant increase in breast and leg muscle in both *MSTN* knockout quail and chicken indicated that *MSTN* can be a potential selection marker for poultry lines with higher meat yield.

In addition to muscle growth, higher feed efficiency is another important trait for economic profit, because feed cost is the major cost in the poultry industry. To improve feed efficiency, partitioning nutrients from fat into muscle is important, and the desirable nutrient partitioning can be achieved by reducing body fat [103]. Body-fat storage is regulated by balancing between lipid synthesis and hydrolysis, and an increase in lipid hydrolysis can decrease overall body fat contents. Lipid hydrolysis is performed by an enzyme called lipase, and adipose triglyceride lipase (ATGL) initiates the breakdown of triglyceride, the main constituents of body fat [104]. ATGL is a rate-limiting enzyme in triglyceride breakdown, and the activity of ATGL is inhibited by the protein encoded by G0/G1 switch gene 2 (*G0S2*) gene [105]. In mice, *G0S2* knockout resulted in enhanced lipolysis [106], whereas G0S2 overexpression inhibited adipose lipolysis in mice and quail [63,107]. Therefore, a *G0S2* knockout chicken, showing decreased abdominal fat deposition, was generated via CRISPR/Cas9, using a PGC-mediated method to investigate lipid metabolism in chickens for potential industrial application in terms of feed efficiency [89].

Like other livestock industries, infectious diseases cause serious problems and financial losses in the poultry industry, and thus, disease resistance is one of the desirable traits in chickens. However, disease resistance is difficult to be obtained by traditional selective breeding because a virus resistant phenotype cannot be compared easily. As an alternative solution, genome editing is actively used to improve disease resistance in the livestock industry [108]. As a first avian model having resistance to a specific infectious disease, avian leucosis virus subgroup J (ALV-J)-resistant chickens were generated by modifying the Na+/H+ exchanger type 1 (*NHE1*) gene, encoding the functional cellular receptor for ALV-J [88,109]. In this study, tryptophan residue number 38 (W38) of chicken NHE1 was precisely deleted by co-transfecting the CRISPR/Cas9 vector and W38 deleted donor template, because W38 is recognized as an important amino acid residue for virus entry [110,111]. Importantly, the same strategy of W38 deletion also can be applied to generate ALV-J resistant turkey, another important poultry species susceptible to ALV-J.

In addition to the broiler industry, domestic chickens are also used for egg production in the layer industry. In the layer industry, the male is unwanted, and thus, male chicks are screened and euthanized immediately after hatching, to reduce raising cost. In addition to the conventional day-old chick culling, in ovo sexing methods have been developed because early sex determination in eggs can reduce costs in hatchery and provide a solution to the ethical problem of chick culling [112,113,114]. In this regard, genetic modification in a sex chromosome can be a future solution, and precise genome editing in Z-chromosome is possible via SSN-mediated homologous recombination, as shown in the DEAD-box helicase 4 gene in Z-chromosome knockout chicken [90]. To apply genome editing in the sex chromosome for in ovo sexing, green fluorescent protein (GFP)-expressing gene cassettes were inserted in the Z-chromosome via CRISPR/Cas9-mediated homologous recombination in chickens [55]. Because the male chicken carries Z- and W-chromosome and the female chicken carries Z- and Z-chromosome, mating of GFP-expressing ZW female with wild-type ZZ male will produce wild-type ZW female and GFP-expressing ZZ male which can be identified and discarded in ovo, using fluorescence-detection devices.

### 4.3. Pharmaceutical Purpose

Protein-based drugs are important sectors in the pharmaceutical industry, and more than 130 protein-based drugs are approved for clinical use by the US Food and Drug Administration (FDA) [115]. Conventionally, bacterial and mammalian cell culture systems have been major methods for the production of protein-based drugs [116]. However, bacterial cell culture systems have limitations on glycosylation, and post-translational modification of recombinant protein and mass production of recombinant proteins from a large-scale mammalian cell culture system are expensive [117,118]. Therefore, production of protein-based drugs from transgenic animals gains attention as a cost-effective alternative to the current cell culture system [119,120]. Among currently reported systems for production of recombinant proteins, such as milk, blood, egg white, seminal plasma, urine, and silk acorn, egg white, has been considered to be the most promising and attractive system, because large amounts of protein in egg white can be continually and non-invasively produced [121]. Chickens especially have several significant advantages as bioreactors, such as large production of eggs per hen, easy mating, short generation time of transgenic lines for scaling up the production, and low maintenance cost. So far, many transgenic chickens have been generated as avian bioreactors by inducing random integration of recombinant protein expression vector into their genomes [122]. However, expressions of recombinant protein in eggs by random integration of transgenes were varied among transgenic chickens [123,124,125]. In addition, ectopic expression of transgene or undesirable insertional mutagenesis caused serious health issues, or even death, in some transgenic chickens [126,127]. To prevent these problems, human interferon β (*hIFN-β*) was precisely integrated into the chicken ovalbumin (*OVA*) locus via CRISPR/Cas9-medaited homologous recombination [54]. As an oviduct-specific gene and the most abundant protein in egg white, *OVA* locus is the most promising locus for sufficient expression of protein-based drugs in egg white, as shown in the abundant expression of hIFN-β in the eggs of the knock-in hen. In addition, chicken eggs have been historically used for influenza-vaccine production [128,129], and allergy-inducing egg white proteins, such as OVA and ovomucoid (OVM), can be a problem in vaccine production, as well as in egg consumption. In this regard, TALENs-mediated knockout of *OVA* and CRISPR/Cas9-meidated knockout of *OVM* in chicken can produce allergen-reduced eggs for potential pharmaceutical and industrial applications [50,51].

## 5. Future Research and Conclusions

For PGC-mediated genome editing in avian species, several methods to isolate avian PGCs have been established [130,131,132]. Subsequently, optimal culture conditions for PGC originated from different avian species have to be established. As the most important poultry species in biology and industry, chickens have been actively used for investigating avian PGCs which lead to generation of GE chickens, using the PGC-mediated method. Although PGC-mediated genome editing is only performed in chickens, PGC cultures are currently available from other avian species, including pheasant, quail, turkey, and duck [133,134,135,136]. Therefore, a knockout or knock-in study from pheasant, quail, turkey, or duck is expected to be reported in the near future. In contrast, the adenovirus-mediated method was recently introduced and is only applied in quail, so far. Nevertheless, this method can be potentially applied to generate various GE avian species for scientific and industrial purposes if adenovirus can deliver target genes to cells of various avian species, in addition to chickens, turkeys, and quail [71,75,76]. For example, GE zebra finch can be a good model to investigate the human language-learning process because of a comparable vocal and language-learning process between songbirds and human [137]. Moreover, the pigeon can be a good model to understand the genetic mechanism behind visual cognition and brain development because of their high visual performance [138]. In addition to genome editing in exogenous or endogenous PGCs via a PGC- or adenovirus-mediated method, respectively, genome editing in the zygote using sperm-transfection-assisted gene editing (STAGE) can be a potential alternative for avian genome editing [139]. After transfection of genome-editing tools into sperm, the transfected sperm are introduced via artificial insemination in hens and induce genome editing in the newly fertilized embryo [140]. Further improvement of STAGE for successful application in various avian species can be beneficial in avian genome editing by generating GE birds in the first generation. As more avian genome-editing methods are available and more avian species are subjected to genome editing, avian research in various fields of biology will be accelerated rapidly.

In addition to the scientific perspective, industrial usage of GE avian species has to be considered. Currently, genome editing in livestock is mainly used to investigate the function of target genes for usage of the target gene as a genetic factor for generating superior lines of livestock with desirable traits. Although commercial usage of GE livestock is not available currently, the FDA approved genetically modified salmon for human consumption for the first time in 2015 [141]. If more GE animals are eventually approved by the FDA, GE poultry will benefit commercial and industrial sectors in the future. In addition to the government regulation, consumer perspectives on GE animals, in terms of ethical and human safety issues, should be carefully addressed [142,143]. In contrast, GE chicken as a bioreactor for production of protein-based drugs in eggs was already approved by the FDA in 2015 [121]. Because it is not for human consumption, and recombinant protein is purified from egg whites for medical usage, the consumer perspective is less intensive. Therefore, performing genome editing in the avian species for direct commercial usage in the biomedical or poultry industry will gain more interest.

So far, the PGC- and adenovirus-mediated genome-editing method has been introduced and applied to only chicken and quail, respectively. However, genome editing in various avian species via a recently developed adenovirus-mediated method or conventional PGC-mediated method is just a matter of time. Although genome editing in the avian species was historically a hard field to improve, currently developed approaches for production of GE birds will significantly advance avian research in the near future.

## Figures and Tables

**Table 1:** Published studies of PGC- and adenovirus-mediated genome editing in avian species.

Species	Mediator	Editing Tool	Purpose	Phenotype	Reference
Chicken	PGC	D10A-Cas9 nickase	Knockout of myostatin gene	Increase of muscle mass	Kim et al., 2020 [87]
Quail	Adenovirus	CRISPR/Cas9	Single amino acid deletion in myostatin propeptide	Increase of muscle mass	Lee et al., 2020 [72]
Chicken	PGC	CRISPR/Cas9-mediated homologous recombination	W38 deletion in Na^+^/H^+^ exchanger type 1	Resistance to leucosis virus subgroup J	Koslová et al., 2020 [88]
Quail	Adenovirus	CRISPR/Cas9	Knockout of melanophilin gene	Exhibition of gray feather color	Lee et al., 2019 [71]
Chicken	PGC	CRISPR/Cas9-mediated nonhomologous end joining repair	Knock-in of GFP into the Z chromosome	GFP expressing progenies for sexing	Lee et al., 2019 [55]
Chicken	PGC	CRISPR/Cas9	Knockout of G0/G1 switch gene 2	Reduction of abdominal fat deposition	Park et al., 2019 [89]
Chicken	PGC	CRISPR/Cas9-mediated homologous recombination	Knock-in of human interferon β into the ovalbumin locus	Production of human interferon β in the egg white	Oishi et al., 2018 [54]
Chicken	PGC	CRISPR/Cas9-mediated homologous recombination	Knockout of immunoglobulin heavy chain variable region (*VH*)	Insertion of a loxP site in the *VH* region	Dimitrov et al., 2016 [80]
Chicken	PGC	CRISPR/Cas9	Knockout of ovomucoid gene	Mutation in ovomucoid gene	Oishi et al., 2016 [51]
Chicken	PGC	TALENs-mediated homologous recombination	Knockout of DEAD-box helicase 4 gene	Sterility in female	Taylor et al., 2017 [90]
Chicken	PGC	TALENs	Knockout of ovalbumin gene	Mutation in ovalbumin gene	Park et al., 2014 [50]
Chicken	PGC	Homologous recombination	Knockout of immunoglobulin light chain locus	Low level of peripherla B cells and antibody	Schusser et al., 2016 [79]
Chicken	PGC	Homologous recombination	Knockout of immunoglobulin heavy chain J gene segment	Lack of peripheral B cells and antibody	Schusser et al., 2013 [49]

PGC: primordial germ cell; DEAD-box: Asp-Glu-Ala-Asp conserved motif; D10A: Asp to Ala substitution in the RuvC domain; CRISPR: clustered regularly interspaced short palindromic repeats; TALEN: transcription activator-like effector nuclease; GFP: green fluorescent protein; VH: immunoglobulin heavy chain variable region.

## Abbreviations

ALV-J	avian leucosis virus subgroup J
ATGL	adipose triglyceride lipase
CRISPR	clustered regularly interspaced short palindromic repeats
DSB	Double-stranded break
EGCs	embryonic germ cells
EGK	Eyal-Giladi and Kochav stage
ESC	embryonic stem cell
F1	the largest preovulatory follicle
F2	the next largest preovulatory follicle
FDA	Food and Drug Administration
G0S2	G0/G1 switch gene 2
GE	genome-edited
GFP	green fluorescent protein
gRNA	guide RNA
HDR	homologous direct repair
hIFN-β	human interferon β
IgH	immunoglobulin heavy chain
IgL	immunoglobulin light chain
Indel	insertion or deletion
LWF	large white follicles
MLPH	melanophilin
MSTN	myostatin
NHE1	Na+/H+ exchanger type 1
NHEJ	non-homologous end joining
OVA	ovalbumin
OVM	ovomucoid
PGC	primordial germ cell
SSNs	site-specific nucleases
STAGE	sperm transfection assisted gene editing
SWF	small white follicle
SYF	small yellow follicle
TALENs	transcription activator-like effector nucleases
W38	tryptophan residue number 38
ZFNs	zinc-finger nucleases
